# RETRACTION: MYH10 Combines with MYH9 to Recruit USP45 by Deubiquitinating Snail and Promotes Serous Ovarian Cancer Carcinogenesis, Progression, and Cisplatin Resistance

**DOI:** 10.1002/advs.202513557

**Published:** 2025-07-26

**RETRACTION**: L. Liu, C. Chen, P. Liu, J. Li, Z. Pang, J. Zhu, Z. Lin, H. Zhou, Y. Xie, T. Lan, Z.‐S. Chen, Z. Zeng, and W. Fang, “MYH10 Combines with MYH9 to Recruit USP45 by Deubiquitinating Snail and Promotes Serous Ovarian Cancer Carcinogenesis, Progression, and Cisplatin Resistance,” *Advanced Science* 10, no. 14 (2023): 2203423, https://doi.org/10.1002/advs.202203423.

The above article, published online on 16 March 2023 in Wiley Online Library (wileyonlinelibrary.com), has been retracted by agreement between the journal Editor‐in‐Chief, Kirsten Severing; and Wiley‐VCH GmbH. The retraction has been agreed upon following an investigation into concerns raised by a third party, which revealed inappropriate image overlaps across multiple figure panels: specifically, between Figures 2B, 6C, and 7C; Figures 2D and 6D; and Figures 2E, 6E, and 7E, representing different experimental conditions. A further review also identified duplicated western blot bands between the Figure panels of 6F and 7F. The authors acknowledged the image misuse, attributing it to the large volume of data collected over the extended duration of the study. They maintain that these errors do not affect the article's conclusions. However, the partial raw data they provided did not adequately explain the discrepancies and was insufficient to verify the integrity of the published results. The number and the level of inconsistencies identified in the published figures raise serious concerns about the handling of the data and the overall integrity of the research. As a result, the editors have lost confidence in the presented data and consider the conclusions compromised. The authors were informed of the retraction decision but did not respond.

